# Preoperative Speech Impairments as Precursors of Posterior Fossa Syndrome in Children with Brain Tumours

**DOI:** 10.1007/s12311-026-02066-3

**Published:** 2026-08-21

**Authors:** Rida Ahmed, Natalie Boll-Avetisyan, Jonathan Kjaer Grønbaek, Ditte Boeg Thomsen, Annet Kingma, Karin Persson, René Mathiasen, Christine Dahl, Andrea Carai, Bianca Andreozzi, Angela Mastronuzzi, Barry Pizer, Colin Thorbinson, Kristian Aquilina, Eelco Hoving, Marianne Juhler, Roel Jonkers, Vânia de Aguiar

**Affiliations:** 1 International Doctorate for Experimental Approaches to Language and Brain (IDEALAB), University of Potsdam, Potsdam, Germany; 2 University of Groningen, Groningen, The Netherlands; 3 Department of Linguistics, University of Potsdam, Potsdam, Germany; 4 Center for Language and Cognition Groningen (CLCG), University of Groningen, Groningen, The Netherlands; 5 Behavioral and Cognitive Neuroscience research school (BCN), University of Groningen, Groningen, The Netherlands; 6 Department of Paediatric and Adolescent Medicine, Rigshospitalet University Hospital, Copenhagen, Denmark; 7 Department of Nordic Studies and Linguistics, University of Copenhagen, Copenhagen, Denmark; 8 Department of Pediatrics/Pediatric Oncology, University Medical Center Groningen, Groningen, The Netherlands; 9 Department of Health Sciences, Lund University, Lund, Sweden; 10 Neurosurgery Unit, Bambino Gesù Children’s Hospital, Rome, Italy; 11 Neurorehabilitation and Adapted Physical Activity Day Hospital, Bambino Gesù Children’s Hospital, IRCCS, Rome, Italy; 12 Department of Hematology/oncology, Cell Therapy, Gene Therapies and Hematopoietic Transplant, IRCCS Bambino Gesù Children’s Hospital, Rome, Italy; 13 Department of Life Sciences and Public Health, Università Cattolica del Sacro Cuore, Milan, Italy; 14 University of Liverpool, Liverpool, UK; 15 Department of Oncology, Alder Hey Children’s NHS Foundation Trust, Liverpool, UK; 16 Department of Neurosurgery, Great Ormond Street Hospital, London, UK; 17 Oncological Pediatric Neurosurgery, Princess Maxima Center for Pediatric Oncology, Utrecht, The Netherlands; 18 Department of Neurosurgery, Aarhus University Hospital, Aarhus, Denmark; 19 Department of Radiation Oncology, University Medical Center Groningen, Groningen, The Netherlands

**Keywords:** Pediatrics, Infratentorial neoplasms, Posterior Fossa Syndrome, Mutism, Speech disorders, Dysarthria, Preoperative period, Risk factors

## Abstract

Preoperative speech deficits in children with posterior fossa tumours (PFTs) may be linked to postoperative speech impairment (POSI), a hallmark of posterior fossa syndrome, yet this relationship remains underexplored. POSI is characterized by mutism or markedly reduced speech that resolves within weeks to months but often leaves persistent motor-speech deficits. Most existing literature has focused on postoperative outcomes, with limited attention to preoperative speech function. We therefore systematically investigated this association by analyzing perceptible motor-speech features in a large cohort of patients scheduled for surgery within the European Study of Cerebellar Mutism Syndrome. We included data from 135 children which formed three age-and sex- comparable groups: 16 patients who later developed POSI, 11 were mute and five had severely reduced speech (3;9–14;4 years), 62 patients with habitual speech (3;2–17;11 years), and 57 healthy controls (3;0–18;1 years). Patients were Italian-, Dutch-, and English-speaking, while controls were Dutch- and Italian-speaking. Four speech-language therapists rated participants’ narrative recordings on 23 speech features (e.g., distorted vowels, imprecise consonants, stuttering, and hoarseness). Group comparisons revealed significant preoperative differences between the children who later developed POSI and the children with habitual speech in both hypernasality and in phonation-respiration (e.g., leaky, hoarse, and strained voice). In addition, patients who later developed POSI showed impairment compared to healthy controls in prosody (e.g., speech rate, excess and equal stress) and articulation (e.g., distorted vowels, imprecise consonants, sequencing errors). Patients with habitual speech differed from the controls in hypernasality and prosody. Moreover, age influenced the severity of articulatory and prosodic symptoms, with younger patients showing greater impairment. Children with PFT show signs of speech impairment preoperatively relative to healthy controls. When comparing patients, those later developing POSI show more severe impairments related to resonance and voice quality. This suggests possible differences in the degree to which speech-related underlying anatomical substrates were compromised, and these early differences may represent potential predictors of later POSI. These findings highlight the value of preoperative assessment for identifying children at a heightened risk of POSI, opening opportunities for prehabilitation - targeted early intervention in the form of speech therapy - to strengthen the speech-motor networks.

**Supplementary Information:**

The online version contains supplementary material available at https://doi.org/10.1007/s12311-026-02066-3.

## Introduction

The posterior fossa is a densely packed region at the base of the skull, containing critical structures, including the cerebellum, brainstem, and fourth ventricle [1]. Around 50% of brain tumours arise in the posterior fossa [2]. Resection of a tumour in the posterior fossa may be followed by an array of neurobehavioural, motor, and linguistic symptoms [3]. These symptoms are collectively referred to as Posterior Fossa Syndrome (PFS) ([4, 5]). A cardinal symptom of PFS is a transient state of mutism (PFS1) ([6] or severely reduced speech (PFS2), limited to single words or to utterances of up to three words. Mutism or severely reduced speech, jointly called postoperative speech impairment (POSI), occur in around 28–30% of patients who undergo tumour surgery, as established by the European study of Cerebellar Mutism Syndrome (CMS) [7]. In these patients, these transient symptoms are often followed by dysarthria, a speech impairment with common symptoms being slow speech rate, monotonous voice, and reduced loudness, which sometimes persist for many years after surgery [8]. Nevertheless, dysarthria has also been reported in children who did not develop mutism after surgery albeit mild [9].

While several studies have addressed dysarthric symptoms in patients *after* a posterior fossa tumour (PFT) surgery, relatively little is known about whether speech in these patients is also impaired *before* the surgery. POSI has almost exclusively been reported as an aftereffect of surgery; however, the presence of the tumour alone can cause speech and language impairments before surgery [10, 11]. Some prior studies have established links between preoperative speech deficits and postoperative mutism [10, 12], but these studies use generic diagnostic labels such as ‘articulation deficit’ to describe preoperative motor-speech impairment without capturing the heterogeneity in the symptoms of speech impairment [13]. Identifying which specific speech features before surgery may predispose patients to POSI is therefore a critical step toward improving risk prediction for POSI.

The current study, therefore, aimed to identify which speech features are precursors to postoperative mutism and reduced speech. For this, preoperative speech samples of patients were perceptually rated and compared with those of healthy controls. We hypothesized that tumour-related disruptions in the posterior fossa, specifically the cerebellum, the brainstem, and the neuronal networks between the cerebellum and the cerebrum, that are implicated in the process of speech production, may cause preoperative speech deficits. We also hypothesize that these deficits may be more pronounced in children who later develop POSI than in children who do not. This work forms part of the multicenter European study of CMS [14], which aims to identify risk factors for PFS by collecting clinical and speech-language data from patients admitted to the collaborating centers across Europe.

### The Posterior Fossa and Speech

The cerebellum is well known for its role in motor control and coordination, and since speech is also a motor process, it is natural that the cerebellum subserves speech-motor control [15]. Speech production is a complex task involving the coordinated activity of various speech subsystems. These subsystems include respiration (pushing air out of the lungs), phonation (vibration of the vocal folds), articulation (movement of the tongue, lips, and jaws), and resonance (the reverberation of the sound across the larynx, pharynx, mouth, and nose; e.g. [16]. These subsystems are controlled by a network of brain regions, among which are the cerebellum and its connections with the cortical speech areas [15]. The cerebellum, specifically, is involved in the muscle coordination, articulation and rhythm of speech [15, 17]. In healthy adults, right cerebellar activation, in combination with activation of the dominant speech areas in the left cerebral hemisphere, has been observed during overt speech tasks such as single-word repetition [18]. Moreover, functional imaging studies show activation of the right cerebellar hemisphere, especially lobules IV, V, and VI, as well as Crus I, during covert speech production tasks such as silent verb generation [19, 20]. Apart from the cerebellar structures, the neuronal network connecting the cerebellum to the cortical speech-motor areas form an important speech circuit. Specifically, the right superior cerebellar peduncle is associated with verbal fluency in neurotypical adults, and speech rate is associated with the middle cerebellar peduncle and the frontal aslant tract [21]. According to the *Directions Into Velocities of Articulators* (DIVA) model, the cerebellum is hypothesized to contribute to the feedforward control subsystem - a speech-motor planning system [22]. Within this subsystem, a cortical network located in the ventral premotor and posterior inferior frontal cortex stores learned speech movements and sends commands to the primary motor cortex for execution. The cerebellum supports this process through cortico-cerebellar loops [22].

The brainstem is another important structure within the posterior fossa which contains cranial nerve nuclei that directly innervate muscles required for respiration, phonation, resonance, and articulation [23]. When damaged, patients experience lower motor neuron weakness, exhibiting in the form of flaccid dysarthria characterized by hypernasality, distorted phonemes, and breathy phonation [13, 23]. Given the importance of the posterior fossa structures in speech-motor control and execution, it is reasonable that lesions in this region would result in speech impairments. The following sections expand on the reports of these speech impairments in PFT patients.

### Speech Deficits after PFT Surgery

Previous studies report deficits in various speech subsystems after surgery for a PFT. Across these studies, articulatory disturbances are most consistently reported, which include, but are not limited to, distorted vowels [8, 13, 24], imprecise consonants [13, 24–26], syllable reduction, and consonant cluster reduction [25]. Earlier case studies show articulation patterns not maturing at the expected rate in children who underwent a PFT surgery [25]. These include persistence of developmental phonological processes such as stopping (substituting a stop sound such as /t/ or /d/ for a fricative or affricate, e.g., saying *‘tun’* for *‘sun’*), post-vocalic devoicing (producing a voiced consonant at the end of a word without voicing, e.g., *‘dog’* pronounced as *‘dok’*), backing to velars (replacing front sounds like /d/ with sounds made further back in the mouth, e.g., *‘gay’* for *‘day’*), progressive assimilation (an earlier sound influencing a later sound in the word, typically in place, manner, or voicing, e.g., the /s/ in “bags” changes to /z/), and regressive assimilation (a later sound influencing an earlier sound in the word e.g., the /n/ in “handbag” changing to /m/ because of the following /b/) [25].

Resonance abnormalities have also been described in the PFT patients after surgery, with hypernasality as most commonly reported symptom [8, 9, 13, 25–27] (but cf. occasional reports of hyponasality) [26, 28]. Inappropriate nasality may arise from velopharyngeal incompetence caused by brainstem tumors [29]. This is reinforced by the study by Van Mourik at al. [13]), which found that a group with brainstem tumours exhibited more frequent hypernasality than a group with cerebellar tumours.

Phonatory deficits are another well-documented outcome after a PFT surgery. These include hoarseness, breathy and/or strained/strangled voice quality [8, 24, 26, 27], hypophonia or reduced loudness [8, 30], excess variation in loudness [24, 31, 32], and explosive vocal onsets [25]. In some cases, these phonatory disturbances are accompanied by deviant respiratory patterns, such as audible inspiration or expiration during speech [9, 27]. Another symptom typical of insufficient breathing support is short phrases, which has also been reported in PFT patients [24, 30]. Furthermore, prosodic disturbances are often reported in children with PFT after surgery including monopitch, abnormal pitch variation, inappropriate stress placement, and excess or equal stress across syllables [8, 25–27, 31].

### Age as a Risk Factor

Young age at diagnosis of a PFT has been associated with a higher risk of speech impairment in terms of mutism and severely reduced speech [6, 7, 33, 34]. However, there are also inconsistencies in the predictive nature of age for the development of mutism [35, 36]. Young age as a risk factor of mutism has been explained by the maturation hypothesis, which states that damage to the reciprocal connections between the cerebellum and the cerebral cortex, that have not yet completely myelinated, results in worse outcomes in younger children [37]. It has also been suggested that symptoms of speech impairment following PFT surgery may differ depending on the age of the patient. For example, the ataxic dysarthria features reported for adults with cerebellar lesions, like scanning speech and irregular articulatory breakdowns, were not found for children with a cerebellar tumour resection [13]. Nevertheless, it remains unclear whether the severity and the type of specific speech symptoms observed in PFT patients depends on the age at diagnosis.

### Speech Deficits Before PFT Surgery

While many studies have focused on postoperative speech deficits, only a few have reported speech function before surgery. The earliest report assessed four children via neuropsychological evaluation [38], and found that postoperative speech deficits resolved in all but one child, who had preoperative dysarthria and cognitive deficits [32]. perceptually analyzed speech function pre- and postoperatively in ten children and found that mild dysarthria was present at both assessment points but was infrequent. Before surgery, one child exhibited signs of ataxic dysarthria, including a mild breathy voice, voice tremor, and alternating loudness. After surgery, three children showed ataxic symptoms, including the same child who had been affected preoperatively. In this case, the same three symptoms persisted, but the breathy voice had worsened in intensity following surgery. It is important to note that this study excluded patients who developed mutism after surgery, limiting our understanding of the preoperative speech profiles of this group. Di Rocco et al. [12] described preoperative language impairment (PLI) in children with PFTs, proposing that PLI is a subclinical state of postoperative mutism, resulting from the presence of the tumour, and exacerbated by surgery. In a subsequent follow-up study, Bianchi et al. [10] expanded this work by adding 36 children and pooling the data from both cohorts. In the combined sample of 70 children, 20 (28.5%) presented with PLI. 90% of the PLI group also presented with motor-speech difficulties in the form of either apraxia of speech (an inability to carry out verbal-motor commands smoothly) or ataxic dysarthria (a cerebellar speech disorder marked by poor timing and coordination). They tested apraxia of speech via orofacial articulatory planning. Among the children with PLI, 70% developed postoperative mutism, 10% had motor-speech impairment immediately after surgery, and 20% showed improvement in their PLI symptoms.

While these studies provide some clear indications that speech may be impaired before surgery, the current knowledge is limited to information on more generic diagnostic labels (dysarthria/apraxia of speech), rather than a specification of impairments across subsystems of speech. In addition, while some case reports provide details on the specific symptoms observed in some individuals, a systematic and detailed observation across a larger group is lacking in the literature for preoperative speech impairments. Since individual speech symptoms can occur across multiple speech disorders and postoperative studies have reported heterogeneous speech symptoms, broad diagnostic labels such as apraxia of speech, flaccid dysarthria, and ataxic speech may obscure clinically meaningful variation that may help determine preoperative precursors of POSI. Additionally, such speech-subsystem level identification of symptoms which can be useful for prehabilitation of specific speech deficits before surgery. Identifying preoperative risk factors of POSI also helps set realistic expectations for the patient and their family regarding the postoperative trajectory. Importantly, such an investigation is a step further in understanding the underlying mechanisms that lead to the development of POSI.

### Aim of the Current Study

The aims of the current study are twofold. Firstly, we want to identify specific subsystems of speech which, when impaired preoperatively, may put patients at a higher risk of developing POSI. Secondly, we want to identify preoperative speech deviances in the two patient groups (children with POSI and habitual speech after surgery) when compared to an age-and-sex matched healthy control group. Apart from the main goals, we also aim to explore whether the age at diagnosis affects the severity of speech impairment, if present, in our PFT sample.

## Method

### Participants

**Fig. 1:**
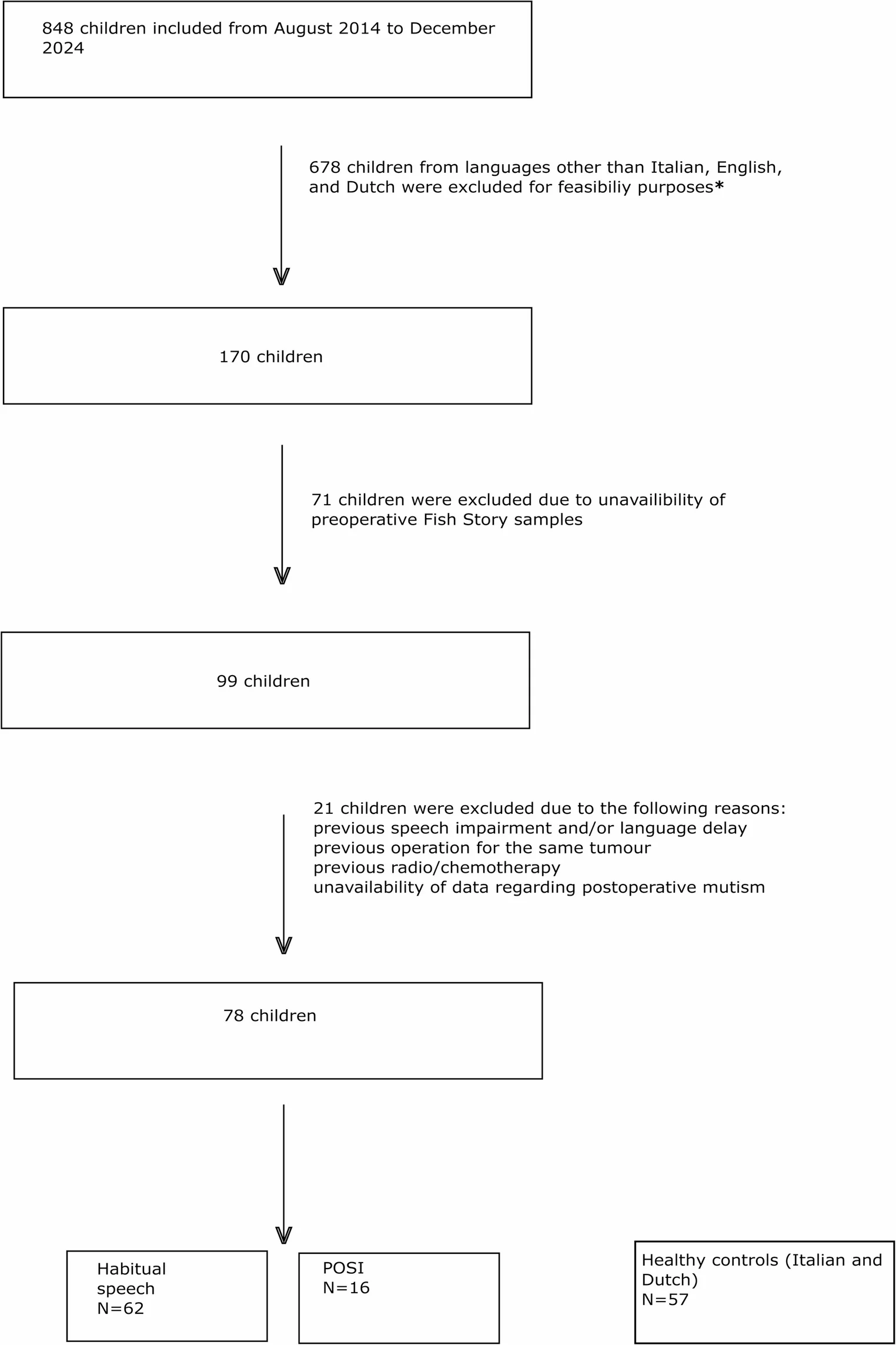
Sample selection. Note. POSI = Postoperative speech impairment *Feasibility reasons included lacking speech language pathologists available to do the perceptual coding across all features and all other languages

A total of 848 children were enrolled in the European Study of CMS [14], a multicenter study aimed at identifying risk factors for CMS by gathering clinical and speech-language data of children who undergo PFT surgery. The study was registered with ClinicalTrials.gov on November 24, 2014 (registration number: NCT02300766). According to the terminology employed in this study, patients who experienced mutism and reduced speech (speech restricted to single words or two-to-three-word utterances) after surgery were categorized as having postoperative speech impairment (*POSI)* [7]. As per the classification of PFS and POSI in previous studies [6, 39], reduced speech was considered a clinical manifestation within the spectrum of posterior fossa syndrome rather than evidence of a distinct neurological entity. Combining both subgroups therefore reflects the current clinical conceptualisation of posterior fossa syndrome while maximising statistical power The inclusion process is outlined in Fig. 1. In our final sample, we include English-, Italian-, and Dutch-speaking participants. Participants who had a pre-existing speech disorder and/or developmental language disorder, or who were already receiving speech therapy, were not included. One participant had a diagnosis of autism spectrum disorder (Asperger syndrome) and another of attention deficit hyperactivity disorder (ADHD); both were included in the study. Although children with autism spectrum disorders or ADHD may have atypical speech profiles, these individuals did not have a documented history of speech and language disorders, and therefore still complied with the inclusion criteria. The included participants formed three groups: (1) a group that later developed POSI (mutism or reduced speech) (*n* = 16), (2) a group that did not develop POSI (*n* = 62), and (3) a healthy control group (*n* = 57). Within the database of the European study of CMS, the patient data were uploaded by the participating centers in each country. The Italian data came from the Bambino Gesu Hospital in Rome. The English data came from multiple centers including Alder Hey Children’s Hospital (Liverpool), Great Ormond Street Hospital (London), and centers in Manchester, Bristol, and Nottingham. Finally, the Dutch data came from participating centers in Groningen, Nijmegen, and Utrecht. Control data were available only for the Dutch (collected at the University of Groningen, Netherlands) and Italian (collected at the Bambino Gesu Hospital, Rome) groups. Details about the included participants and the speech data are provided in Table 1.

**Table 1:** Information about participants and data

Participant groups	Languages	Age range Mean (SD) years; months	Number of females (%)	Tumour types	Number of utterances Mean(SD)	Number of words Mean(SD)
POSI (*n* = 16)	English (*n* = 6) Italian (*n* = 7) Dutch (*n* = 3)	3;9–14;4 9;1 (3;7)	6 (37.5%)	Medulloblastoma (50%) Pilocytic astrocytoma (19%) Ependymoma (6%) Other (12.5%) NA (12.5%)	19 (5.56)	111 (55.7)
Habitual speech (*n* = 62)	English (*n* = 32) Italian (*n* = 11) Dutch (*n* = 19)	3;2–17;11 10;1 (3;4)	22 (35.5%)	Medulloblastoma (19.5%) Pilocytic astrocytoma (58%) Ependymoma (6.5%) Other (10%) NA (6%)	18.3 (5.25)	125 (54.2)
Healthy control (*n* = 57)	Italian (*n* = 25) Dutch (*n* = 32)	3;0–18;1 9;3 (3;8)	20 (35%)	-	21.6 (6.22)*	150 (31.8)*

*POSI *= Postoperative speech impairment, *SD* = Standard deviation

* Two missing values

### Procedure

#### Speech Recordings

We used audio recordings of assessments of the children’s narrative skills. For this, an examiner showed the pictures from the Fish Story within the *Expression*,* Reception*,* and Recall of Narrative Instrument (ERRNI*) [40], and the participants narrated a story based on these pictures. The participants were audiorecorded while they narrated the story, and the examiner provided minimal feedback such as “okay”, “good job”, “what do you see here”, and so on. The speech data were collected within approximately three days before the date of surgery.

##### Perceptual Speech Ratings

Four speech-language therapists were recruited to perceptually rate the speech characteristics of the participants. Each rater evaluated only recordings in a single language: one native Dutch speaker rated all Dutch samples, one native Italian speaker rated all Italian samples, and two non-native but highly proficient English speakers rated English samples. The native languages of the two English raters were Turkish and Italian. One of the English raters rated all samples while another re-rated 38 (28% of the total sample) to establish inter-rater reliability. They were asked to listen to the recordings of the narrative production of participants and rated, for each patient’s recording, 23 speech features (e.g., distorted vowels, imprecise consonants, stuttering, and hoarseness, see Appendix A) on a scale from 0 to 3, with 0 indicating no impairment (i.e., absence of a specific feature), and 3 indicating severe impairment. The selected speech features capture a range of motor-speech difficulties spanning ataxic dysarthria (e.g., features related to articulatory imprecision, irregular prosody such as excess and equal stress, voice and resonance difficulties, and reduced phonatory-respiratory coordination) [41], and apraxia of speech (e.g., features related to motor-speech planning such as irregular articulatory breakdowns, sequencing errors, consonant cluster segmentations, and self-corrections [42]. Moreover, stuttering was included in the list of speech features as it has been correlated with the disruption of white matter fibres in the cerebellar peduncles [43]. While rating the speech samples, colloquial use of speech that fell outside the standard variety was not considered a deviation. For instance, in Dutch, the devoicing of /v/ is common and therefore was not rated as a deviation for the feature deviant voicing. For Italian, the weakening of /ʎ/ to /j/ was not classified as an imprecise consonant when it was evident from the overall recording that the pronunciation aligned with dialectal speech patterns. The inter-rater reliability analysis revealed variable agreement between raters across the 23 perceptual features. Weighted kappa values ranged from − 0.04 to 0.72. There was substantial agreement for stuttering (κw = 0.72, percent agreement = 89.47)), imprecise consonants (κw = 0.67, percent agreement = 60.52), slow rate (κw = 0.65, percent agreement = 68.42), and irregular articulatory breakdowns (κw = 0.64, percent agreement = 89.47). Several features showed moderate agreement (e.g., consonant assimilation (κw = 0.55, percent agreement = 78.94), prolonged phonemes (κw = 0.55, percent agreement = 63.15), leaky voice (κw = 0.55, percent agreement = 57.89)), while monopitch (κw = 0.13, percent agreement = 27.0), self-correction (κw = 0.25, percent agreement = 92.10), short phrases (wk = 0.29, percent agreement = 42.10), and deviant voicing (wk = 0.29, percent agreement = 68.42) had slight agreement. The lowest agreement was for consonant cluster segmentation (κw = -0.04, percent agreement = 84.21). Such variability in the inter-rater reliability for different speech features is a recurring issue in subjective perceptual ratings and has been reported widely in previous studies [44]. Even though some measures have low weighted kappa values but high percent agreement, we consider weighted kappa as our primary measure of inter-rater reliability, as it considers the ordinal nature of the ratings. Ratings from the two English raters for 28% of the total sample were summarized using the median and interquartile range (IQR) and are presented in Appendix C. The median rating was 0 for most speech features across both raters, indicating that these features were absent in at least half of the observations. Variability was generally low, although some features (e.g., prolonged intervals, slow rate, and short phrases) showed greater dispersion as evidenced by a larger IQR. It should be noted though that this sample contained only six patients from the POSI group and 32 patients from the habitual speech patient group. The mild nature of impairment in the habitual speech group may be the reason why medians for most speech features were 0. Supplementary Fig. 2 represents the frequency of rating per speech feature by the two raters.

### Data Preprocessing and Analysis

**Fig. 2:**
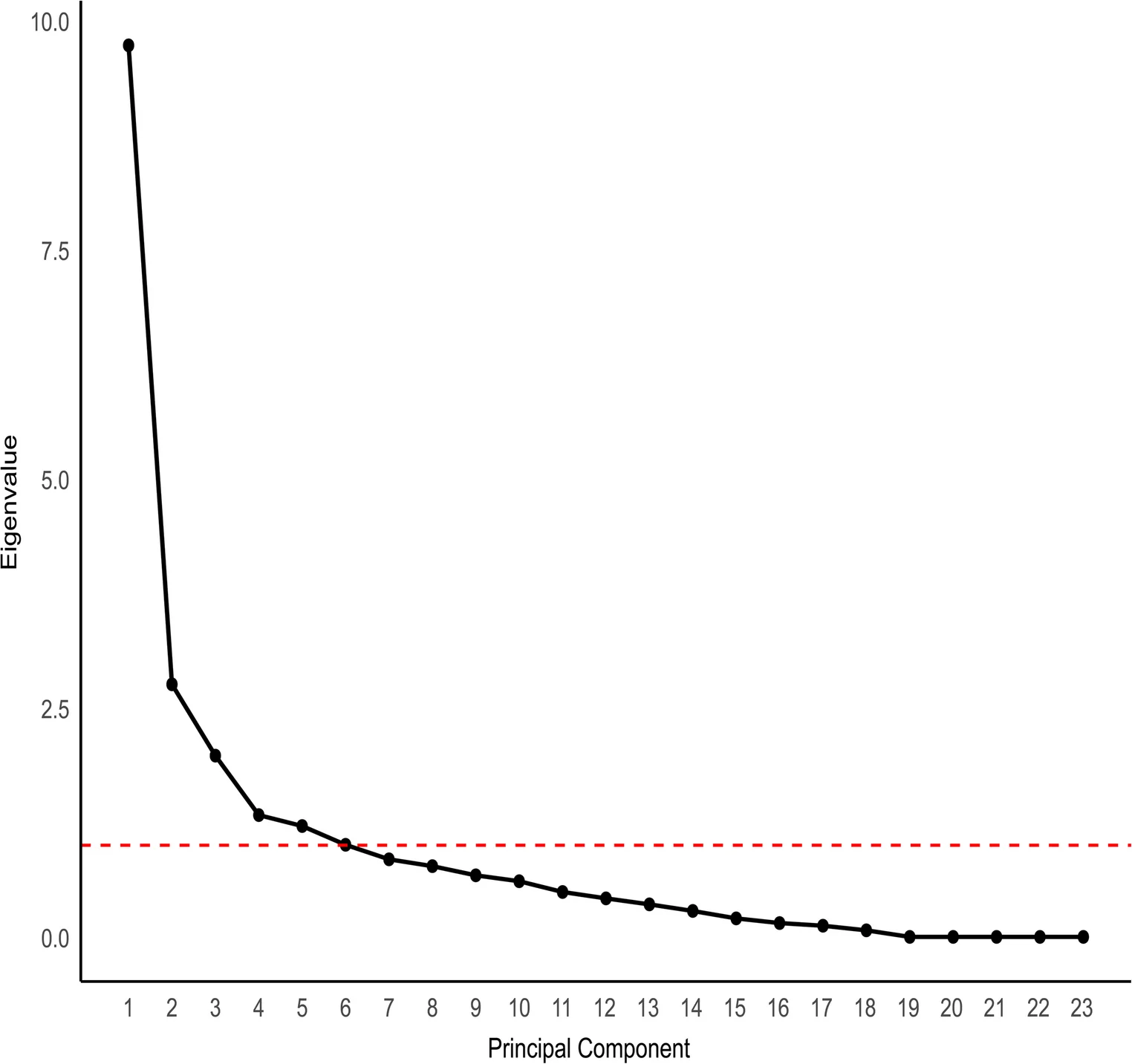
Screeplot

To reduce dimensionality and enhance the interpretability of the results, we performed a principal component analysis (PCA) on the ratings of all 23 variables for all participants (pooling patients and controls). Before running the PCA, the ratings were z-scaled (standardized to have a mean of 0), and a polychoric correlation matrix was computed among all variables to capture the ordinal nature of the perceptual ratings. The PCA was then conducted using the varimax rotation, which helps the variables load more strongly onto one component rather than moderately onto multiple components, hence aiding interpretability. Afterward, the scree plot (Fig. 2) of the eigenvalues was examined, and five components with eigenvalues greater than one were retained following Kaiser’s criterion [45]. These five components explained 74% of the total variance in the data. Speech features with a loading ≥ 0.5 were considered to meaningfully contribute to the components [46]. Refer to Table 2 for the loading matrix.

**Table 2:** Loadings of speech variables on extracted principal components

Variable	RC1	RC2	RC3	RC4	RC5
	Articulation	Phonation-Respiration	Speech fluency	Prosody	Hypernasality
Distorted vowels	**0.804354**	0.028163	0.099306	-0.03046	0.029868
Imprecise consonants	**0.601398**	0.031483	0.286239	0.146226	0.390128
Consonant assimilation	**0.685454**	0.034194	0.21238	0.230404	0.066176
Deviant voicing	0.380147	0.213986	0.341718	0.274725	0.415178
Consonant cluster segmentation	0.29301	-0.05348	0.161533	**0.816294**	0.207154
Consonant cluster reduction	**0.745887**	0.063108	-0.10743	0.391566	0.295452
Omitted syllables	**0.667564**	-0.11917	0.023719	0.400828	0.272226
Omitted phonemes	**0.717376**	0.182136	-0.15101	0.320097	0.255431
Sequencing errors	**0.792673**	0.295194	0.128824	0.009371	-0.06104
Irregular articulatory breakdowns	**0.781862**	0.159677	0.203719	0.346487	0.103763
Self-correction	0.152704	-0.0533	**0.857522**	-0.08984	-0.0013
Stuttering	0.092776	0.003604	**0.859612**	0.306276	-0.00186
Excess and equal stress	0.307392	0.417657	0.18396	**0.724475**	0.076089
Prolonged phonemes	**0.588707**	0.212057	0.156549	0.470652	-0.17941
Prolonged intervals	**0.68498**	0.380696	0.335715	0.244066	-0.12863
Slow rate	**0.525237**	0.44831	-0.21931	**0.588784**	-0.21187
Monopitch	0.343294	**0.520414**	0.056975	0.291457	0.234172
Reduced loudness	0.364197	**0.797742**	-0.13592	0.032589	0.1077
Leaky voice	-0.09803	**0.813766**	0.239846	0.2335	0.158557
Hoarseness	-0.02119	**0.809596**	-0.06225	-0.1587	0.049313
Strained voice	0.167611	**0.739764**	-0.01336	0.47314	0.108009
Hypernasality	0.0957	0.326358	-0.03806	0.058629	**0.82016**
Short phrases	0.453021	0.478398	0.483701	0.006807	0.214526

Bold values indicate meaningful loadings

Several clear components emerged representing impairments of individual speech subsystems. The first component was primarily defined by features related to articulation, with strong loadings from distorted vowels, imprecise consonants, consonant assimilation, consonant cluster reduction, omitted syllables, omitted phonemes, sequencing errors, irregular articulatory breakdowns, slow rate, and prolonged phonemes/intervals. The second component was characterized by meaningful loadings from reduced loudness, leaky voice, hoarseness, strained voice, and a moderate loading from monopitch. This component captured dysfunctions in phonation and respiration. The third component represented speech dysfluency as it was driven by self-corrections and stuttering. The fourth component was characterized by prosodic difficulties such as excess and equal stress and slow rate, along with one articulatory feature, consonant cluster segmentation. Finally, the fifth component had a single strong loading from hypernasality.

Slow speech rate was the only feature with crossloadings on articulatory and prosodic components. This pattern likely reflects motor control processes with similar perceptual consequences, where a slow speech rate can emerge fundamentally from prosodic timing disruption but may also represent a compensatory strategy for articulatory imprecision. Two features, namely, deviant voicing and short phrases, did not load significantly onto any single component. This may reflect the multidimensional nature of deviant voicing, as it may be caused by articulatory or phonatory difficulties. We expected short phrases, occurring due to insufficient support from the respiratory system, to load onto the respiratory component. However, the loadings did not reflect this, which may indicate that this feature was not sufficiently consistent across participants to cluster within a specific subsystem. Deviant voicing and short phrases were also among the features with the lowest inter-rater reliability; therefore, the inability to cluster into any speech component may have been a result of the unreliability of their ratings.

After examining the component loadings, component scores were computed as weighted averages by multiplying the loadings matrix by the standardized ratings [47]. This step yielded scores of all five components for each participant, representing the extent to which each participant exhibited impairments in each subsystem. To enable further analyses, component scores were merged with demographic and clinical data, including age, sex, and participant group.

After the preprocessing step, a multivariate analysis of covariance (MANCOVA) was conducted to examine group differences across multiple outcome variables. We included ‘group’ as the independent variable with three factor levels (POSI, habitual speech, and healthy), and sex and age as covariates, as these can explain some variability in speech production patterns. Five speech components were included as dependent variables, and Pillai’s Trace test was used to check for the effect of group, sex, and age, since it is more robust to unequal sample sizes. Afterward, separate univariate analyses of covariance (ANCOVAs) were conducted to determine which specific speech components contributed to the overall multivariate effect. P-values from these ANCOVAs were adjusted for multiple comparisons using the Benjamini-Hochberg false discovery rate procedure. To identify which specific group contrasts drove significance, post-hoc pairwise comparisons were performed with Tukey adjustment to account for multiple testing.

To investigate how age affected speech component scores differently across groups, we followed up with Analyses of Variance (ANOVAs) with Age*Group interaction. Afterward, separate linear models were fitted for the significant interactions, and age-related slopes of groups were compared using emtrends. All analyses were conducted in R Studio [48].

As a sensitivity analysis, the PCA was repeated after excluding the five speech features with weighted kappa values < 0.40. The resulting five-component solution was highly consistent with the original analysis. The articulation, speech fluency, and hypernasality components were preserved, while redistribution of loadings was observed within the phonation–prosody components, including the emergence of hoarseness as a distinct component. These findings suggest that the overall PCA structure was somewhat robust to the exclusion of variables with lower inter-rater reliability. Afterward, all subsequent analyses, including the MANCOVA, univariate ANCOVAs and pairwise comparisons were repeated. The results of these analyses are described in Appendix B. Moreover, since control data were not available for the English-speaking group, we ran a sensitivity analysis by excluding the English patients (See Appendix B).

## Results

The overall MANCOVA indicated a significant multivariate effect of group (*Pillai’s Trace* = 0.24, *F* (10, 252) = 3.45, *p* < .001) and age (*Pillai’s Trace* = 0.11, *F* (5, 125) = 3.22, *p* = .009) on component scores as a whole, but not of sex (*Pillai’s Trace* = 0.05, *F* (5, 125) = 1.18, *p* = .321). Following up, univariate ANCOVAs for each speech component revealed significant group differences, after Benjamini-Hochberg correction, for principal components representing features related to articulation (*F* (2, 129) = 4.20, *p* = .036, η² = 0.05), phonation-respiration (*F* (2, 129) = 7.37, *p* = .003, η² = 0.10), prosody (*F* (2, 129) = 6.86, *p* = .004, η² = 0.09), and hypernasality (*F* (2, 129) = 11.27, *p* < .001, η² = 0.14 ). No significant group effect was observed for the fluency component (*F* (2, 129) = 2.15, *p* = .154, η² = 0.03). Age was a significant covariate for symptoms related to articulation (*F* (1, 129) = 13.86, *p* = .002, η² = 0.10, fluency (*F* (1, 129) = 5.91, *p* = .036, η² = 0.04), and prosody (*F* = 12.74, *p* = .002, η² = 0.09). Age did not significantly affect phonation-respiration (*F* (1, 129) = 3.3, *p* = .118, η² = 0.02), and hypernasality (*F* (1, 129) = 3.6, *p* = .112, η² = 0.03). Moreover, sex was not a significant predictor of any of the component scores (all *p* ≥ .15).

**Fig. 3:**
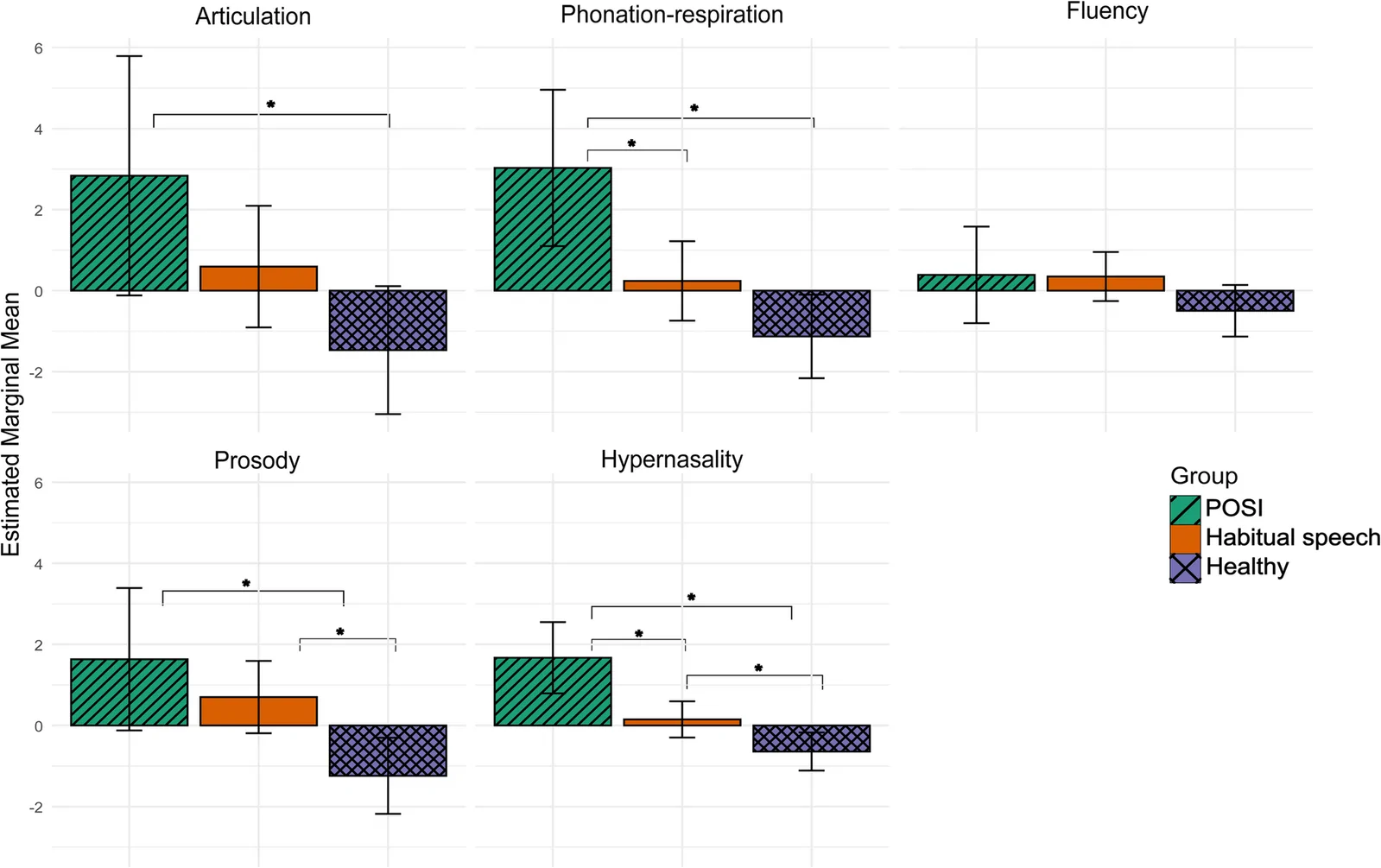
Estimated marginal means of speech components (y-axis) by group (x-axis). Note. POSI stands for postoperative speech impairment (i.e., mutism and/or reduced speech). A separate visualization of individual component score distributions is provided in Supplementary Figure 1

To clarify which groups differed from each other on each speech component, post-hoc Tukey-adjusted comparisons were conducted. These revealed that the POSI and habitual speech groups differed in phonation-respiration (*p* = .032) and hypernasality (*p* = .008). Moreover, the POSI and healthy control group also differed in phonation-respiration (*p* < .001) and hypernasality (*p* < .001), and additionally they differed in articulation (*p* = .032) and prosody (*p* = .014). Lastly, (just like the POSI group) the habitual speech and healthy control group differed in hypernasality (*p* = .045) and prosody (*p* = .010). No other group comparisons reached significance. Table 3 presents all post-hoc results, and Fig. 3 shows the estimated marginal means of each group by speech components.

**Table 3:** Results of Tukey-adjusted pairwise comparisons between participant groups across speech components

Speech component	Contrast	Estimate	SE	t-value	*p*-value(Tukey-adjusted)
Articulation	POSI – Habitual speech	2.24	1.67	1.34	0.376
	POSI – Healthy	4.30	1.69	2.54	**0.032**
	Habitual speech – Healthy	2.06	1.10	1.87	0.151
Phonation-Respiration	POSI – Habitual speech	2.79	1.09	2.55	**0.032**
	POSI – Healthy	4.16	1.11	3.76	**< 0.001**
	Habitual speech – Healthy	1.37	0.72	1.90	0.142
Prosody	POSI – Habitual speech	0.93	1.00	0.94	0.618
	POSI – Healthy	2.88	1.01	2.86	**0.014**
	Habitual speech – Healthy	1.94	0.66	2.97	**0.010**
Hypernasality	POSI – Habitual speech	1.52	0.50	3.05	**0.008**
	POSI – Healthy	2.31	0.50	4.59	**< 0.001**
	Habitual speech – Healthy	0.79	0.33	2.42	**0.045**
Fluency	POSI – Habitual speech	0.04	0.67	0.06	0.099
	POSI – Healthy	0.88	0.68	1.29	0.399
	Habitual speech – Healthy	0.84	0.44	0.90	0.141

**Fig. 4:**
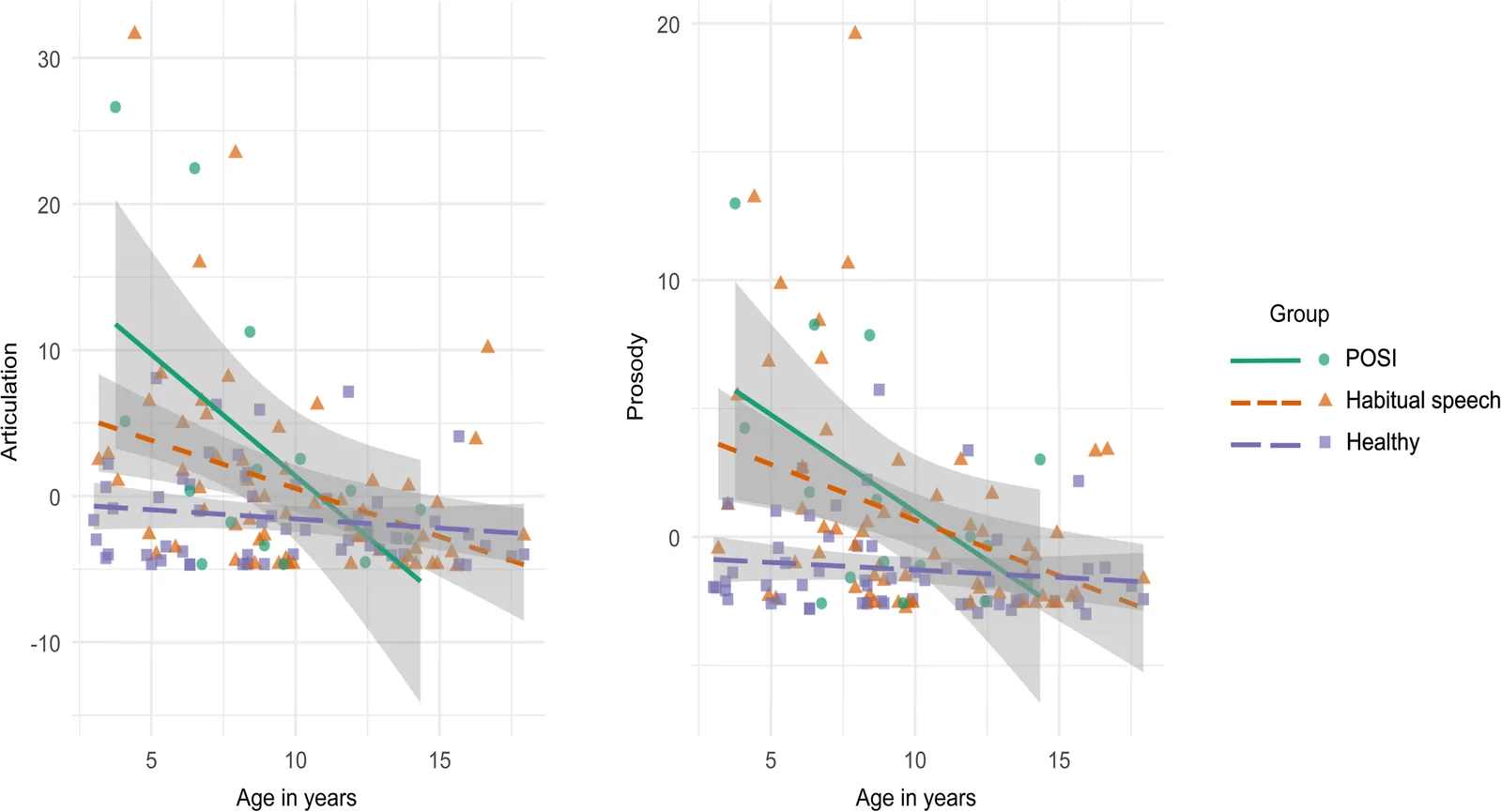
Group specific linear relationships between age and speech impairment for articulation (left) and prosody (right). Note. The x-axis represents participants' age in years, the y-axis represents a continuous score representing speech symptoms where a higher score means more severe impairment related to articulation (left) and prosody (right), and each slope represents the group-specific regression between age and speech component score. POSI stands for postoperative speech impairment (i.e., mutism and/or reduced speech).

Since age was a significant covariate for symptoms related to articulation, fluency, and prosody, we conducted follow-up univariate analyses only for these components. For each of these components, we ran an ANOVA including the Age*Group interaction to determine whether the relationship between age and component scores differed across groups. This interaction was significant for symptoms related to articulation (*F* (2, 127) = 5.93, *p* = .011, η² = 0.09) and prosody (*F* (2, 127) = 4.58, *p* = .029, η² = 0.07) after Benjamini-Hochberg correction. To explore these interactions, we fitted separate linear models for the two components and used *emtrends* to estimate and compare age-related slopes between groups. These comparisons revealed that for articulation, the age slope in POSI patients was significantly more negative than in healthy controls (estimate = − 0.126, *p* = .004), indicating that younger patients had more severe ratings for articulation symptoms. For prosody, the POSI group also showed a significant slope difference compared to healthy controls (estimate = − 0.058, *p* = .043), and the habitual speech group showed the same trend (estimate = − 0.031, *p* = .049). This means that the prosodic symptoms were more severe in younger patient groups. No other slope contrasts reached significance. Figure 4 shows the age slopes of three participant groups for articulatory and prosodic symptoms.

## Discussion

The current study aimed to identify signs of preoperative speech impairments in different speech-subsystems in children with PFTs and explore whether some of these signs could serve as distinguishing features between the POSI and habitual speech patient groups. When compared to healthy controls, the POSI group showed more signs of speech impairment across all speech subsystems, except fluency. The habitual speech group, on the other hand, showed increased hypernasality and disrupted prosody compared to healthy controls. Importantly, the POSI group had significantly worse ratings for hypernasality and phonation-respiration components than the habitual speech group.

### Auditory-Perceptual Precursors to POSI

The POSI group showed greater hypernasality compared to the habitual speech group and the healthy control group. In previous literature, hypernasality has commonly been reported in children with PFTs after surgery [9, 13, 25–27], and one study found it exclusively for the patient group that had recovered from mutism [8]. Since hypernasality is often a result of damage to the cranial nerves that control velopharyngeal function, its incidence has been related to brainstem involvement of the tumour [13]. Furthermore, preoperative compression of the brainstem by the tumour has been associated with a higher incidence of mutism postoperatively [49]. This may suggest that hypernasality is a behavioural manifestation of brainstem compression, which is a preoperative risk factor for POSI. In our sample, out of 16 patients who later developed POSI, 11 had some degree of hypernasality (two were rated severe, six were rated moderate, and 3 were rated as mild). Five of these patients had a tumour in the fourth ventricle; two in the brainstem, three expanding both in the fourth ventricle and cerebellar vermis; and one had a tumour that extended to the brainstem, vermis, and both cerebellar hemispheres, thereby assuming that part of the tumour was also located in the fourth ventricle. This suggests that patients in the POSI group may have had either direct brainstem compression, in cases where the tumour was located within or extended into the brainstem, or secondary brainstem compression, where tumours in adjacent structures such as the fourth ventricle or cerebellum exerted mass effect on the brainstem. Such direct or indirect compression may have clinically presented as the lack of velopharyngeal control with hypernasal speech.

Next, the phonation-respiration component showed a significant difference between the two patient groups before surgery. This speech component included features such as reduced loudness, leaky voice, strained voice, hoarseness, and monopitch. This pattern is in line with previous reports of harsh and strained voice quality observed exclusively in the mutism group after surgery [8]. These features, resembling a profile of flaccid dysarthria, are a result of insufficient control of the respiratory and phonatory apparatus, which control the pushing of air out of the lungs (i.e., expiration), coordinated with laryngeal motor control and aerodynamics which cause the vibration of vocal folds in the larynx [50]. Breathing specific to speaking is controlled by a network of brain structures involving the brainstem, cerebellum, and postcentral sulcus, as shown by an fMRI study focused on adult speech production in the context of respiratory control [51]. The study showed that the brainstem respiratory nuclei generate automatic breathing patterns and integrate sensory inputs to regulate air exchange. Moreover, the cerebellum has bidirectional connections with both brainstem respiratory nuclei and cortical regions responsible for respiratory control, and acts as a coordinating center [51]. A tumour causing damage to the brainstem and/or the cerebellum may disrupt these functions. With regards to the vocal fold vibrations, branches of the vagus nerve (cranial nerve X) originating in the brainstem, innervate the laryngeal muscles [52], which are important for voice production. Reduced functionality of these nerves due to compression by the tumour could result in the symptoms described above.

Lastly, in articulation, the POSI group showed significant impairment when compared to the healthy controls, while this pattern was not present for the patient group who did not later develop POSI. The articulation component consists of symptoms related to speech planning (i.e., sequencing errors) and execution (i.e., distorted vowels, imprecise consonants, consonant assimilation, slow rate among others). Previous research has reported articulation impairments, including prolonged phonemes, syllable reduction, imprecise consonants, and irregular articulatory breakdowns [24, 25, 30], in children who developed mutism after surgery, and the same may be true for these patients before surgery, albeit in a less severe form. An auditory-perceptual study of speech after PFT surgery found imprecise consonants, prolonged phonemes, and irregular articulatory breakdowns only in the patient group who had developed mutism after surgery [8]. Distorted vowels were present in both groups but were more frequent in the group that had recovered from mutism (71% vs. 30% in the group without mutism). It has been postulated that lesions of cranial nerves V, VI, X, and XII affect articulation [23], which may help explain the articulatory deficits observed preoperatively in the POSI group, particularly given that these patients already exhibited other symptoms suggestive of cranial nerve involvement. Cranial nerve assessment was performed preoperatively in our patient sample and within the POSI group, three out of the total 16 patients exhibited impairment of cranial nerves. One patient presented with bilateral impairment of CN II, another with right-sided impairment of CN VII, and a third with left-sided impairment of CN VI. Aside from speech impairment caused by compression or damage to the cranial nerves, tumour infiltration of the cerebellar hemispheres themselves may also contribute to speech deficits (e.g., previous studies suggest that the lobule VI/lobulus simplex of the cerebellar hemispheres is involved in movements of lip, face and tongue [49, 50]). In our POSI sample, four patients had tumours affecting the cerebellar hemispheres.

The distribution of tumour types differed between patient groups, raising the possibility that tumour-related factors could contribute to differences in preoperative speech characteristics. When tumour type was included as a covariate in a sensitivity analysis restricted to patient participants, it did not significantly explain variation in the principal component scores, and the group effect remained significant. That is, we did not find evidence for (or against) the possibility that the observed differences between patient groups are substantially attributable to tumour type distribution alone. Nevertheless, tumour type may represent a proxy for differences in neuroanatomical location, tumour extent, or involvement of speech-related networks, and these factors could not be fully disentangled in the current sample. Future studies with larger samples and more balanced tumour-type distributions are needed to further investigate the contribution of tumour-specific characteristics to preoperative speech profiles.

### Speech Symptoms distinguishing Patients from Controls

Both the POSI and the habitual speech group exhibited significantly more severe preoperative speech symptoms than healthy controls in both hypernasality and prosody (with prosody reflecting loadings from slow rate, excess and equal stress, and consonant cluster segmentation). These findings are consistent with earlier reports of post-operative slowed, irregular speech rhythm and resonance abnormalities in patients with cerebellar tumours [24–26]. Importantly, the only speech domain that did not show significant differences among any pair of groups was fluency (reflecting loadings from stuttering and self-correction). These were also infrequent symptoms, with 10% of the patients presenting with self-corrections and 14% with stuttering [8]. also found that phoneme repetition (i.e., one of the hallmarks of stuttering) was not present in any patient group, either with or without mutism. It is important to note that speech impairments in prosody and hypernasality were also present in the patient group that did not later develop POSI, but to a lesser extent than in the POSI group. In the POSI group, all subsystems showed worse scores than healthy controls, except for fluency. Previous studies also attest to such graded nature of speech impairment in the PFT patient group after surgery as they found dysarthria in patients who did not develop mutism after surgery, although the incidence was low in this group compared to the group who was recovering from mutism [9, 53].

Previous studies that observed preoperative speech and language deficits have suggested that a subclinical state of impairment exists already before surgery, which is exacerbated by surgery and manifests itself in more pronounced deficits afterward [10]. These preoperative disruptions may result from several factors: Firstly, there may be direct cerebellar damage due to tumour infiltration, which can disrupt the functioning of cerebellar lobules implicated in speech production [19]. Secondly, if the tumour is located in or close to the brainstem, it can compress the cranial nerves, leading to a lack of velopharyngeal control, presenting as resonance deficits [54], and/or damage to the hypoglossal nerve innervating tongue muscles, leading to articulation deficits [55]. Thirdly, the presence of a tumour may result in the obstruction of the flow of cerebrospinal fluid, leading to hydrocephalus, which has been shown to result in neuromotor speech deficits in children born with cerebellar dysmorphology [56]. Finally, a tumour in and around the cerebellum may cause cerebello-cerebral diaschisis [57], a phenomenon in which cerebellar damage may induce reduced functioning in structurally intact cortical speech-motor areas via the disrupted cerebello-thalamo-cortical pathways. However, the literature on cerebello-cerebral diaschisis in PFT patients mostly refers to the surgical damage [57, 58], and not to the tumoural damage to the surrounding tissue, which happens over an extended time period. Since we observe speech impairments already before surgery in the current study, a future study could carry out preoperative functional imagining to confirm whether there is presurgical hypoperfusion of the cerebral areas in the presence of cerebellar tumours.

### The Influence of age on the Degree of Speech Impairment

Age was a significant factor in determining the severity of symptoms related to articulation and prosody, albeit for some group contrasts only. Firstly, younger patients in the POSI group showed more severe articulation symptoms than the older patients, when their age trend was compared to healthy controls. Previous studies have reported such a disadvantage of young age with regards to the incidence of mutism [6, 7, 33, 34], but these studies did not explore whether specific speech subsystems were more impaired in younger than in older children. Secondly, the prosodic speech symptoms were more severe in younger patients compared to older patients for both the POSI and the habitual speech group, when the age trend was compared to healthy controls. This shows that both patient groups had an age-related disadvantage regarding prosodic symptoms. It has been hypothesized that the incomplete maturation of the connections between the cerebellum and the motor-speech related cortical regions can make younger children more prone to worse functional outcomes due to a lesion [37]. Our results are compatible with this maturation hypothesis.

### Limitations and Future Directions

The current study is the first step of systematically investigating preoperative speech-subsystem level impairments in children with PFTs and identifies the speech levels most likely to appear as precursors of POSI. At the same time, the results also point to several avenues for future research. Firstly, although perceptual analysis of speech as a method is a useful method and the clinical gold-standard to gauge perceptible speech impairments, one has to consider the subjectivity of the ratings. Our data were rated by four raters, each of whom may have had a slightly different way of perceiving impairment severity. This becomes even more relevant given that the Italian- and Dutch-speaking raters rated both patients and controls (giving them a relative idea of impairment severity), whereas the English rater only rated the patients.^1^ This limitation can be addressed by measuring the relevant speech features directly from the speech signal via acoustic analysis. For example, the current study’s result of hypernasality and voice quality being the distinguishing features between the two patient groups can be confirmed with extracting the A1-PO (the difference between the first oral formant and the nasal resonance peak) as a measure of nasality [59] and cepstral peak prominence as a measure of voice quality [60]. Since we also found deviant speech features in patients who did not develop POSI, albeit to a less severe degree, future confirmatory acoustic analyses will be useful in quantitatively assessing this graded nature of speech impairment. Moreover, raters were aware of whether speech recordings originated from patients or healthy controls, although they were blinded to the distinction between the POSI and habitual speech patient groups. Consequently, comparisons between patient subgroups can be considered relatively unbiased, whereas comparisons between patients and controls may be subject to bias and should therefore be interpreted with caution and require further validation. A further limitation concerns the relatively small effect size observed for speech fluency. This suggests that any group differences in this domain may be subtle, and the absence of statistically significant findings should therefore be interpreted with caution. Replication in larger, independent samples is warranted to determine whether these findings are robust and to better characterize potential differences in speech fluency among children with PFTs.

The preoperative precursors of POSI were symptoms related to the phonatory-respiratory and resonance subsystems. This finding can be useful in prehabilitative approaches to strengthening the speech networks responsible for these functions in the brain. Non-invasive prehabilitation in the form of neuromodulation coupled with intensive speech therapy has reportedly yielded positive results in adult patients with supratentorial tumours [50–52]. To implement such programs for children, extensive research is needed, first on the neural mechanisms of different speech processes and then on the feasibility of neurostimulation-aided speech therapy for children. Moreover, to investigate the anatomical correlates of speech impairment in children with PFTs, the scores on speech features in the current study can be correlated with the lesion location using voxel-based lesion symptom mapping. This will not only inform about the neuro-anatomical source of speech impairments in our patient population but also inform about the localisation of speech function in healthy individuals. Together, these follow-up studies are important for translating the present findings into clinically relevant applications, including the development of predictive tools and providing neurosurgeons with guidance regarding eloquent regions of the cerebellum. Lastly, our findings pave the way for future prediction studies, which will help set realistic expectations concerning post-surgical outcomes, and enable providing counselling to the patients and their caregivers regarding their postoperative trajectory.

## Conclusion

In summary, our study showed that speech-subsystem level impairments are already perceivable in children with posterior fossa tumours before surgery. The study also attests to the graded nature of speech impairment in patients with a PFT, showing that a marked impairment profile before surgery may be an early sign that there is preoperative compression/damage to the anatomical substrates, especially to those of phonatory and velopharyngeal control for patients who develop POSI. Such systematic reports of speech impairments were limited in previous literature where general diagnostic labels were applied to the PFT patients’ speech profiles based on heterogeneous symptoms and hence the anatomical sources of the impairment could not be hypothesized. We also confirmed that older age at diagnosis has a preventive role, specifically against articulatory and prosodic impairments. The present findings add to the existing literature by showing that coordinated speech-motor control is affected rather than isolated speech features, generalizable across different languages. This finding may guide speech-language pathologists to target the affected speech subsystems in a child, potentially enabling interventions that improve multiple speech features associated with a given subsystem, thereby supporting more generalizable treatment outcomes.

## Ethical Considerations

The current study used patient data from the European Study of CMS. The Research Ethics Committees of the Capital Region (H-6–2014–002) in Denmark gave their approval for the collection of data for this project, and the study was authorized in the UK afterwards.

## Supplementary Information

Below is the link to the electronic supplementary material.

Supplementary Material 1 (DOCX 36.6 KB)

Supplementary Material 2 (DOCX 715 KB)

## Data Availability

The data used in this research are part of the European Cerebellar Mutism Study and were shared with researchers from the University of Groningen for the purposes of this study. Data requests should be sent to the PI of the European Cerebellar Mutism Study (Rene.Mathiasen@regionh.dk).

## Footnotes

1 Control data were unavailable for English speakers.
