# Supplementary material for: Preoperative Speech Impairments as Precursors of Posterior Fossa Syndrome in Children with Brain Tumours
Source: Cerebellum. 2026 Aug 21;25(5):130. doi: 10.1007/s12311-026-02066-3 (PMC13493439; doi:10.1007/s12311-026-02066-3)
Supplement: Supplementary file 2 [file 12311_2026_2066_MOESM2_ESM.docx]

Supplementary figure 1

Distribution of individual component scores

POSI stands for postoperative speech impairment (i.e., mutism and/or reduced speech). The horizontal line shows the median component score for each group.

Supplementary figure 2

Distribution of perceptual ratings assigned by each rater across speech features

Note. Stacked bar plots showing the frequency of perceptual ratings assigned by Rater 1 (left panel) and Rater 2 (right panel) for each speech feature. Data are from 38 English speaking patients.
