# Supplementary material for: Preoperative Speech Impairments as Precursors of Posterior Fossa Syndrome in Children with Brain Tumours
Source: Cerebellum. 2026 Aug 21;25(5):130. doi: 10.1007/s12311-026-02066-3 (PMC13493439; doi:10.1007/s12311-026-02066-3)
Supplement: Supplementary file 3 [file 12311_2026_2066_MOESM1_ESM.docx]

# Appendices
## Appendix A
Included speech features and their description
No.
Speech feature
Description (and example, if necessary)
Video examples (if publicly available)
1.
Distorted vowels
Vowel sounds are so deviant that it is difficult to understand the child. What counts as a deviant vowel sound depends on the language examined and how much variation in vowel pronunciation is licensed in everyday speech.
https://www.youtube.com/watch?v=03VfMujZrfw
2.
Imprecise consonants
Consonants may be slurred, indistinct, or poorly articulated. The production may include incomplete closures (imprecise stops or fricatives), distorted place or manner of articulation, or overlapping or merging of consonantal features.
https://www.youtube.com/shorts/4GV_tt--gD4
3.
Consonant assimilation
Consonants that are not well articulated because they are affected by their neighbouring phoneme, typically in place, manner, or voicing, in order to ease articulation. For example, the /s/ in "bags" changes to /z/.

4.
Deviant voicing
Incorrect use of voicing or aspiration. Example: saying "pat" with a voiced /p/ sound, making it sound like “bat."

5.
Consonant-cluster segmentation
Breaking up a consonant cluster into separate sounds. 
Example: pronouncing "spoon" as "s-poon."

6.
Consonant-cluster reduction
One or more consonant sounds in a cluster (a sequence of two or more adjacent consonants within a word) are omitted
Example: pronouncing "spoon" as “poon."

7.
Omitted phonemes
One or more phonemes are omitted from a word.
Example: pronouncing “cat” as “ca”. 

8.
Omitted syllables
One or more phonemes are omitted from a word. 
Example: pronouncing broccoli as bcoli

9.
Sequencing errors
Misordering of sounds in a word. Example: "animal" pronounced as "aminal."

10.
Irregular articulatory breakdowns
Unlike consistent errors - where the same sound is always produced incorrectly - irregular breakdowns are nonsystematic breakdowns in accuracy of articulation. It affects different sounds at different times and may change within or across utterances, resulting in variable speech intelligibility.
Example: Red truck:
wed tuck
Wed duck
Red duck
Red truck 

11.
Self-correction
Correcting the mispronounced word:
Danny saw a wed truck, a red truck 

12.
Excess and equal stress
There is excess stress on usually unstressed parts of speech (such as unstressed syllables in polysyllabic words, e.g. grammatical endings, or monosyllabic function words such as articles, pronouns and auxiliaries), leading to equal stress. Without the normal alternation between stressed and unstressed syllables, the perceptual impression is monotonous.
Note that the child can, of course, stress usually unstressed parts of speech for pragmatic reasons of emphasis (e.g., ‘SHE ‘ate the ‘cake instead of She ‘ate the ‘cake or We ‘HAD ‘crossed the ‘bridge instead of We had ‘crossed the ‘bridge). Such stress used for pragmatic emphasis is normal and does not count as excess and equal stress.

13.
Prolonged phonemes
There are prolongations of phonemes.
This is a motor-speech parameter. If it’s clear that the child hesitates for semantic reasons, e.g., searching for words or wondering how to describe a scene, this hesitation does not count as exhibiting prolonged phonemes.

14.
Prolonged intervals 
Prolonged intervals between syllables and/or words within the same utterance.
Long pauses between utterances do not count as prolonged intervals.
This is a motor-speech parameter. If it’s clear that the child hesitates for semantic reasons, e.g., searching for words or wondering how to describe a scene, this hesitation does not count as exhibiting prolonged intervals.

15.
Slow rate 
The child speaks noticeably slowly, e.g., because of prolonged phonemes, prolonged intervals or a combination of both. (The child’s overall speech rate may also be characterized as slow, even if neither phonemes nor intervals on their own are characterized as prolonged).
Slow rate is assessed per intuition, not by measuring segments per second.

16.
Monopitch

The voice sounds monotone, staying at one pitch level. It may sound like a depressed voice. The pitch level may also be unusual (too high or too low), suggesting that the child is not able to control pitch flexibly.

17.
Reduced loudness
Insufficient loudness. The child does not achieve normal voice loudness and speaks with too low a volume.
https://iowaprotocols.medicine.uiowa.edu/protocols/slide-05d-bilateral-vocal-cord-paralysis-dysphonia-and-dysphagia
18.
Leaky voice 
Air escapes during phonation, creating a breathy sound, similar to whispering.

19.
Hoarseness
A hoarse voice is characterized by roughness and rasp. It can carry both strained and breathy qualities. It is caused by vocal fold irregularities.


https://www.youtube.com/watch?v=zhXbqCp0oac
20.
Strained voice 
A tight, tense, or squeezed voice due to excessive muscular tension, especially around the glottis. In its most severe degree, the voice sounds strained or strangled as if the child squeezes out speech through the glottis with effort.

https://www.youtube.com/watch?v=1gqAGauWj4U
21.
Hypernasality 
Excessive nasal resonance, causing non-nasal phonemes to be produced with a nasal feature.
https://www.youtube.com/watch?v=KWz5_fpnZYc
22.
Short phrases (insufficient coordination between respiration and phonation)
Phrases are short because the child cannot control airflow while speaking and runs out of air. It may sound as if the child is out of breath; there may be more inspirations than you would expect, there may be gasps at the end of phrases, and the child may speak while inhaling.

Note that Short phrases is NOT a grammatical measure in this study. A child may speak in short phrases for grammatical or other reasons, but to score a Yes on this parameter, there must be manifest audible cues that the child speaks in short phrases due to insufficient coordination between respiration and phonation.
https://www.youtube.com/watch?v=zhXbqCp0oac
23.
Stuttering
Stuttering is an abnormally high frequency and/or duration of disfluencies in speech. These disfluencies usually take the form of (a) repetitions of sounds, syllables, or one-syllable words, (b) prolongations of sounds, or (c) “blockages” of airflow or voicing. If any of these disfluencies occur in the sample, it will be regarded as stuttering.


## Appendix B
## Supplementary analyses
### Reanalysis after excluding speech features with low inter-rater reliability
Since five speech features had low inter-rater reliability (i.e., weighted kappa <0.40), we performed a sensitivity analysis by excluding these features from the PCA structure. The articulation and hypernasality components remained similar, while the features from the prosody component (i.e., excess and equal stress, and slow rate) now clustered together with the phonation-respiration component. Hoarseness loaded onto a component of its own with a loading of 0.84. The revised component scores were then entered into the MANCOVA and subsequent analyses. The overall multivariate group effect remained significant. Significant group differences were retained for articulation, phonation–prosody (previously phonation-respiration), and hypernasality, whereas fluency (now consisting only of stuttering) remained non-significant. The principal component consisting only of hoarseness did not show significance. Age continued to significantly predict articulation, phonation–prosody, and fluency, and the age-by-group interaction for articulation remained significant. The previously observed age-by-group interaction for prosody (combined with phonation-respiration features) was no longer significant. Following Benjamini–Hochberg correction, the articulation group effect showed a small reduction in statistical strength (raw p = .022; adjusted p = .055), although the pattern of pairwise comparisons remained unchanged, with the POSI and healthy control groups continuing to differ significantly.
### Sensitivity analysis excluding English speaking participants
To address potential bias due to the absence of English-speaking healthy controls, all patient-vs-control analyses were repeated after excluding English-speaking participants and combining the POSI and habitual speech patient groups into a single patient group. The overall pattern of findings was consistent with the primary analyses. Patients showed significantly higher adjusted scores than healthy controls for phonation–respiration (p = .031), prosody (p = .017), and hypernasality (p = .005). Speech fluency remained non-significant (p = .112), and the difference in articulation was attenuated and no longer statistically significant (p = .078), although the estimated group difference remained in the same direction as in the primary analysis.
### Sensitivity analysis excluding patients with reduced speech from the POSI group
To establish the robustness of the decision to consider the mutistic and the reduced speech group as a unified POSI group, sensitivity analysis was performed in which all multivariate and follow-up analyses were repeated after excluding the five patients with reduced speech. The findings were highly consistent with the primary analyses, with no changes in the pattern of statistically significant group differences. Specifically, significant differences between POSI and healthy controls were retained for articulation (estimate = 4.30 in the original analysis vs. 5.54 in the new analysis; adjusted p = .032 vs. .038, respectively), phonation-respiration (estimate = 4.16 vs. 4.48, adjusted p = .032 vs. .015), prosody (estimate = 2.88 vs. 3.36, adjusted p = .014 vs. .013), and hypernasality (estimate = 2.31 vs. 2.54, adjusted p < .001 in both analyses). Likewise, significant differences between POSI and habitual speech patients remained for phonation-respiration (2.79 vs. 3.11, adjusted p = .032 in both analyses) and hypernasality (estimate = 1.52 vs. 1.75, adjusted p = .008 vs. .006), while the habitual speech and healthy comparisons remained significant only for prosody (estimate = 1.94 in both analyses, adjusted p = .010 vs. .009) and hypernasality (0.79 in both analyses, adjusted p = .045 vs. .034). All non-significant comparisons remained non-significant. Notably, exclusion of the reduced speech subgroup did not attenuate the observed effects; if anything, several p-values were smaller after exclusion.
### Patient group analysis including language
As a sensitivity analysis, language was included as an additional covariate in a patient-only MANCOVA model to evaluate whether differences between languages contributed to variation in perceptual speech characteristics. Language showed a significant multivariate effect on the combined principal component scores (Pillai’s trace = 0.446, F(10,130) = 3.73, p < .001). In follow-up ANCOVAs, language showed nominal effects for articulation (F = 3.41, p = .039), prosody (F = 3.29, p = .043), and hypernasality (F = 4.42, p = .016); however, these effects did not remain significant after Benjamini–Hochberg correction (all p > .05). Importantly, after controlling for language, group differences remained significant for the multivariate outcome. It is important to note that the factors language and rater were confounded in our sample, since each rater rated each language

## Appendix C
Table 1
Descriptive statistics of two English raters for 38 patient samples

Rater 1
Rater 2
Feature
Median
IQR
Median
IQR
Distorted vowels
0
0
0
1
Imprecise consonants
0
1
0.5
2
Consonant assimilation
0
0
0
0.75
Deviant voicing
0
0.75
0
0
Consonant cluster segmentation
0
0
0
0
Consonant cluster reduction
0
0
0
0
Omitted phonemes
0
0.75
0
1.75
Omitted syllables
0
0
0
0.75
Sequencing errors
0
0
0
0
Irregular articulatory breakdowns
0
0
0
0
Self correction
0
0
0
0
Stuttering
0
0
0
0
Excess and equal stress
0
1
0
1
Prolonged phonemes
0
1
0
1
Prolonged intervals
0
0.75
0
2
Slow rate
0
1
0
2
Monopitch
0
0
1
1.75
Reduced loudness
0
1
1
1
Leaky voice
0
0.75
1
1
Hoarseness
0
0
0
0
Strained voice
0
1
0
0.75
Hypernasality
0
0
0
1
Short phrases
0
0.75
1
2
